# Targeted Metabolomics of Tissue and Plasma Identifies Biomarkers in Mice with NOTCH1-Dependent T-Cell Acute Lymphoblastic Leukemia

**DOI:** 10.3390/ijms25126543

**Published:** 2024-06-13

**Authors:** Valeria Tosello, Ludovica Di Martino, Erich Piovan

**Affiliations:** 1 Basic and Translational Oncology Unit, Istituto Oncologico Veneto IOV—IRCCS, 35128 Padova, Italy; valeria.tosello@iov.veneto.it; 2 Dipartimento di Scienze Chirurgiche, Oncologiche e Gastroenterologiche, Università di Padova, 35128 Padova, Italy; ludovica.dimartino@studenti.unipd.it; 3 UOC Immunologia e Diagnostica Molecolare Oncologica, Istituto Oncologico Veneto IOV—IRCCS, 35128 Padova, Italy

**Keywords:** T-cell lymphoblastic leukemia, metabolism, NOTCH1 signaling, leukemia growth

## Abstract

While the genomics era has allowed remarkable advances in understanding the mechanisms driving the biology and pathogenesis of numerous blood cancers, including acute lymphoblastic leukemia (ALL), metabolic studies are still lagging, especially regarding how the metabolism differs between healthy and diseased individuals. T-cell ALL (T-ALL) is an aggressive hematological neoplasm deriving from the malignant transformation of T-cell progenitors characterized by frequent NOTCH1 pathway activation. The aim of our study was to characterize tumor and plasma metabolomes during T-ALL development using a NOTCH1-induced murine T-ALL model (ΔE-NOTCH1). In tissue, we found a significant metabolic shift with leukemia development, as metabolites linked to glycolysis (lactic acid) and Tricarboxylic acid cycle replenishment (succinic and malic acids) were elevated in NOTCH1 tumors, while metabolites associated with lipid oxidation (e.g., carnitine) as well as purine and pyrimidine metabolism were elevated in normal thymic tissue. Glycine, serine, and threonine metabolism, glutathione metabolism, as well as valine, leucine, and isoleucine biosynthesis were enriched pathways in tumor tissue. Phenylalanine and tyrosine metabolism was highly enriched in plasma from leukemia-bearing mice compared to healthy mice. Further, we identified a metabolic signature consisting of glycine, alanine, proline, 3-hydroxybutyrate, and glutamic acid as potential biomarkers for leukemia progression in plasma. Hopefully, the metabolic differences detected in our leukemia model will apply to humans and contribute to the development of metabolism-oriented therapeutic approaches.

## 1. Introduction

T-cell acute lymphoblastic leukemia (T-ALL) is an aggressive hematological neoplasia arising from the malignant transformation of developing T-cells [1]. T-ALL represents approximately 15% of pediatric and 25% of adult ALL cases. T-ALL, with respect to B-ALL, is historically linked with a poor prognosis [2]. The advent of high-dose polychemotherapy regimens has increased survival; however, the outcome of relapsed and chemo-resistant T-ALL still remains poor. Thus, a better understanding of the pathogenesis, more sensitive diagnostic tools, and novel and less toxic therapeutic strategies are required. In cancer, metabolic homeostasis is lost due to the heightened demand for metabolites necessary for growth and proliferation [3]. Metabolomics is an emerging field linked to systems biology that can comprehensively analyze global, dynamic, and endogenous metabolites [4]. Not surprisingly, there has been interest in finding biomarkers that can predict the likelihood or occurrence of relapse in a timely manner, or that represent novel targets for innovative therapies [5,6]. Although metabolomics has previously been used to investigate metabolic differences between ALL patients (mainly B-cell ALL) and normal volunteers [7,8], in vivo data supporting metabolic changes of leukemic cells in pediatric ALL are limited. Metabolomic studies using clinical serum/plasma samples further face numerous challenges due to variability in metabolite concentrations linked to various genetic and environmental factors. Additionally, serum/plasma samples from newly diagnosed leukemia patients may not be easily available. Oncogenic mutations can directly alter cellular metabolism in a cell-intrinsic way, with microenvironmental cues such as hypoxia, nutrient availability, and crosstalk from neighboring cells also affecting cancer metabolism. In T-ALL, NOTCH1 activating mutations are present in over 60% of cases and strongly affect the metabolism in these cells [9]. NOTCH1 facilitates leukemic cell growth through direct transcriptional regulation of numerous anabolic pathways, including ribosome biosynthesis, protein translation, and nucleotide and amino acid metabolism [10,11] and increased expression of MYC, a direct NOTCH1 target [12]. Numerous mouse models of NOTCH1-induced T-cell leukemia have been described [13], where high levels of activated NOTCH1 in murine T-cell progenitor models impair T-cell maturation, leading to the accumulation of CD4+CD8+ double-positive (DP) cells, promoting thymic-independent T-cell development, and ultimately leading to T-cell leukemia closely resembling human T-ALL. Given the many similarities between mouse and human metabolism [14], we took advantage of a mouse model of NOTCH1-induced T-cell leukemia to gain important insight into the metabolomics of T-ALL and to identify putative metabolite biomarkers useful for diagnosis and prognosis.

## 2. Results

### 2.1. Metabolite Profiling in Leukemic Tissue

**Figure 1:**
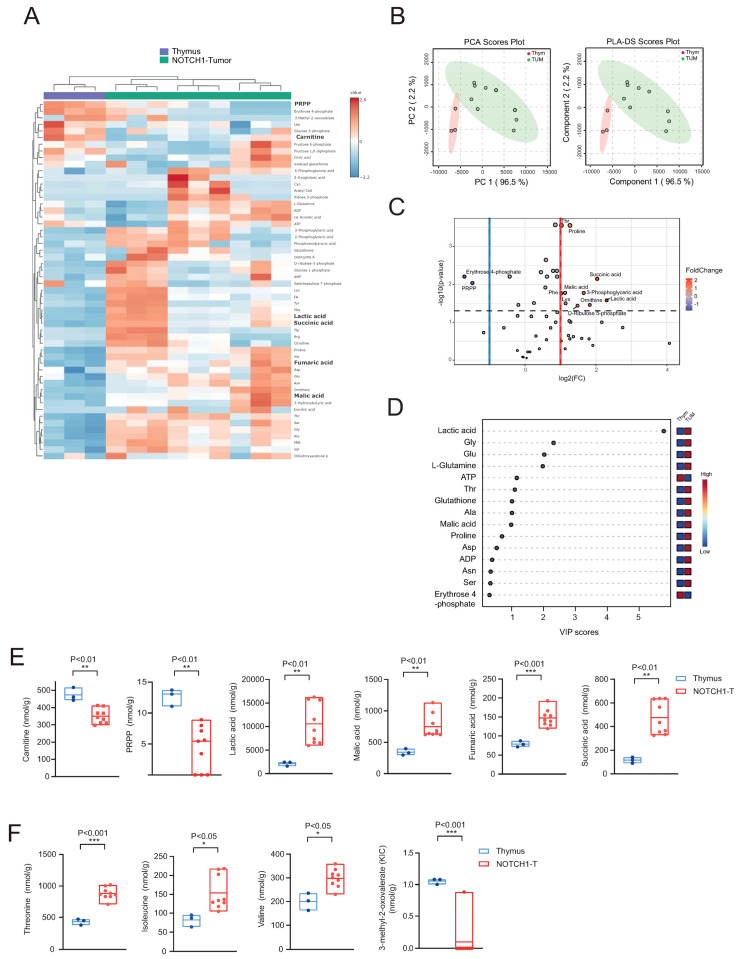
(**A**) Heat map representation of metabolites identified by capillary electrophoresis time-of-flight mass spectrometry (CE-TOFMS) in healthy thymuses of C57BL/6 mice (n = 3) and ΔE-NOTCH1 tumors (n = 9; three different transplantation experiments). Some differentially expressed metabolites are highlighted in bold. (**B**) Principal component analysis (PCA; left) and partial least squares-discriminant analysis (PLS-DA; right) plots. (**C**) Volcano plot showing differentially expressed metabolites between normal thymic tissue (Thym; >80–90% DP cells) and ΔE-NOTCH1 tumors from spleens (TUM; >80–90% DP cells). Only metabolites with a false discovery rate (FDR)-corrected *p*-value < 0.05 and 0.5 ≤ log2 fold change (FC) ≥ 2 are shown. (**D**) PLS-DA variable importance in projection (VIP) score derived from metabolite profiling data of normal thymus versus ΔE-NOTCH1 tumors. (**E**,**F**) Floating bars showing F-scope metabolic quantification for selected metabolites differentially expressed between thymic tissue and ΔE-NOTCH1 tumors. Floating bars represent min and max values; solid lines represent the mean. Significance was calculated using an unpaired *t*-test. * *p* < 0.05, ** *p* < 0.01, *** *p* < 0.001.

To determine the metabolic changes contributing to NOTCH1-dependent T-ALL development, we analyzed a well characterized NOTCH1-induced murine T-ALL model (Δ*E-NOTCH1*-induced model of T-ALL). In this model, overexpression of an activated form of NOTCH1 lacking a major portion of the extracellular domain (Δ*E-NOTCH1*) in transplanted Lin-negative murine hematopoietic cells leads to T-ALL (NOTCH1-T tumors) [13]. Interestingly, the development of T-ALL involves an early phase characterized by the rise of an abnormal, non-tumorigenic polyclonal CD4+CD8+ double-positive (DP) subset at 2–3 weeks of transplantation and a late phase characterized by the rise in a highly tumorigenic, monoclonal DP leukemic population at 6–10 weeks after transplantation. We initially compared the metabolic profile of thymic tissue from healthy 6–7-week-old C57/BL6 mice (where >90% of cells are DP) and infiltrated splenic tissue (NOTCH1-T tumors; >90% of cells are DP) from leukemic mice. We quantified 56 metabolites by using a highly sensitive capillary electrophoresis-time-of-flight mass spectrometry (CE-TOFMS) and capillary electrophoresis–tandem mass spectrometry (CE-MS/MS) method. Hierarchical clustering analysis represented in a heatmap (Figure 1A) and principal component analysis (PCA; Figure 1B) illustrate clear sample clustering or separation between normal and leukemic tissue. Partial least squares-discriminant analysis (PLS-DA) accentuated the separation (Figure 1B). The results of the cross-validation (Q2 = 0.84; R2 = 0.74; accuracy = 1.0) and the permutation testing (*p* = 0.037 for 2000 permutations) revealed that the PLS-DA model was of good and predictive quality, with the separation found not due to chance.

The analysis revealed 10 upregulated and 2 downregulated metabolites (false discovery rate (FDR)-corrected *p*-value < 0.05 and 0.5 ≤ log2 fold change (FC) ≥ 2) in NOTCH1-T tumors compared to thymic tissue (Figure 1C and Supplementary Table S1). The PLS-DA variable importance in projection (VIP) score, which ranks metabolites on the basis of their capacity to separate groups, identified eight metabolites with a VIP score > 1 (including lactic acid, glycine, glutamate, alanine, glutathione, and threonine; Figure 1D).

While thymic tissue preferentially expressed metabolites associated with lipid oxidation (e.g., carnitine) as well as purine and pyrimidine metabolism (PRPP), metabolites found elevated in NOTCH1-T tumors were linked to glycolysis (lactic acid) and TCA cycle replenishment (succinic, fumaric, and malic acids), indicating a significant metabolic shift with the development of leukemia (Figure 1E). NOTCH1-T tumors were also characterized by increased concentrations of some branched chain amino acid (BCAA) pathway metabolites (Figure 1F).

**Figure 2:**
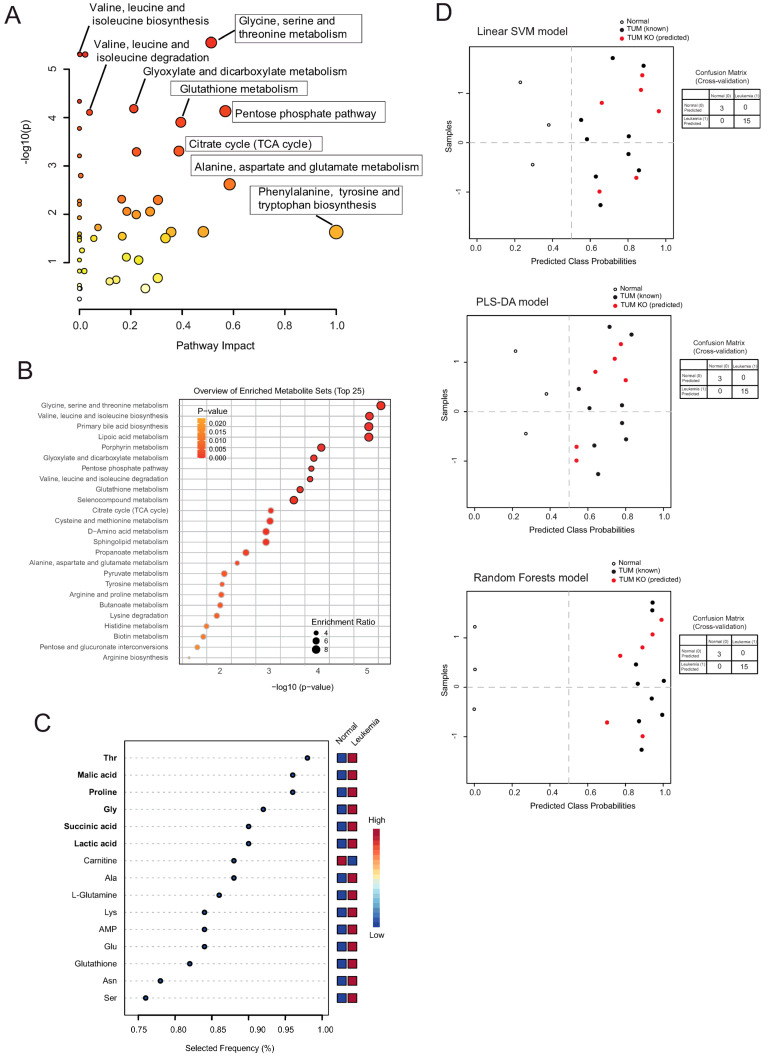
(**A**) Bubble chart depicting Metabolomics Pathway Analysis (MetPA) generated by MetaboAnalyst 6.0 software, using the KEGG database. The color of each circle represents *p*-values from pathway enrichment analysis (Y-axis; darker colors indicate more significant changes of metabolites in the corresponding pathway). On the other hand, circle size relates to the pathway impact score from pathway topology analysis (X-axis). Thus, node color and radius are based on the *p*-value and pathway impact value, respectively. (**B**) Metabolite Set Enrichment Analysis (MSEA) was used to determine differentially enriched metabolite sets between normal thymic tissue and ΔE-NOTCH1 tumors. The top 10 enriched pathways are highlighted with bordered circles. (**C**) The multivariate exploratory ROC analysis (PLS-DA classification method, SVM feature ranking method) was used to identify the best biomarker candidates between ΔE-NOTCH1 tumors (infiltrated spleens; Leukemia) and healthy thymic tissue (Normal). The ranked feature details table is depicted. (**D**) Evaluation of biomarker models based on six identified biomarker metabolites (threonine, malic acid, proline, glycine, succinic acid, and lactic acid) was undertaken using different algorithms: linear support vector machine (SVM; top); PLS-DA (middle); random forests (bottom). These models were applied to samples used for building the model (known; empty circles and black circles) and unknown samples to predict (red circles). Averages of predicted class probabilities for each sample in the 100 cross-validations are summarized in the prediction overviews. Corresponding confusion matrices are provided next to each prediction overview.

Metabolic pathway analysis (MetPA) was used to identify major metabolic pathways that were altered following leukemic transformation. Thus, all metabolites were uploaded to the web-based MetaboAnalyst 6.0 tool for pathway analysis. MetPA identified numerous metabolic pathways altered following leukemic transformation, including phenylalanine, tyrosine, and tryptophan biosynthesis (impact score = 1.0), pentose phosphate pathway (impact score = 0.56), glycine, serine, and threonine metabolism (impact score = 0.51), arginine biosynthesis (impact score = 0.48223), glutathione metabolism (impact score = 0.39476), and citrate cycle (TCA cycle; impact score = 0.38729) (Figure 2A; Supplementary Table S2). Further, BCAA metabolism as well as glycine, serine, and threonine metabolism were identified as significantly enriched pathways in NOTCH1-T tumors by metabolite set enrichment analysis (Figure 2B).

We subsequently attempted an exploratory biomarker analysis to identify potential biomarkers that distinguish neoplastic tissue (leukemia) from the putative normal tissue of origin (thymus). As mentioned above, our analysis revealed altered BCAA levels following leukemic transformation. Further, BCAAs have been implicated in sustaining T-cell activation [15], with changes in BCAA metabolism in different cancers being largely due to BCAT1 overexpression. BCAT1 is the cytosolic enzyme commonly responsible for the reversible transfer of an amino group from leucine, isoleucine, and valine (BCAAs) to alpha-ketoglutarate (α-KG) to form glutamate and the corresponding α-ketoacid (BCKA) [16]. Bcat1 has limited expression in adult tissues, while it is often highly expressed in many tumors [17]. Analyzing gene expression data from a similar NOTCH1-induced murine T-ALL model (where overexpression of an activated, intracellular form of Notch1 (ICN1) in transplanted Lin-negative murine hematopoietic cells leads to the development of an abnormal DP T cell subset at 2 weeks of transplantation followed by the rise of a highly tumorigenic DP leukemic population at 6–8 weeks of transplantation [18]), we found Bcat1 to be highly upregulated in leukemic DP cells compared to normal DP cells. The increase in Bcat1 expression occurred early in T-ALL development, as shown in Supplementary Figure S1A. Thus, we thought Bcat1^−/−^ NOTCH1-T tumors could manifest distinct metabolic features to Bcat1^+/+^ NOTCH1-T tumors, somewhat mimicking metabolic heterogeneity found in human disease and may be an interesting model to include to evaluate our metabolic classifier for leukemia. To achieve this aim, we analyzed the predictive performance of six metabolites identified to be consistently upregulated in leukemic tissue (infiltrated spleen) compared to normal tissue (thymus) using the PLS-DA classification method (Figure 2C). The predictive performance of our selected biomarker candidates (*n* = 6) based on the obtained area under the ROC curve (AUROC) is illustrated in Supplementary Figure S2. Targeting of the *Bcat1* gene and generation of the total null alleles using CRISPR/Cas9 were performed by Cyagen (USA) using the depicted targeting strategy (Supplementary Figure S1B,C). As expected, Bcat1 protein was not detectable in tissues of Bcat1 KO mice known to express Bcat1, such as the brain, kidney, and ovary (Supplementary Figure S1D). Transduced bone marrow (BM) progenitor cells (GFP+Lineage-cKit+Sca1+) from Bcat1 KO were transplanted into lethally irradiated C57BL/6J hosts. There was robust engraftment of ΔE-NOTCH1-transduced cells at two to three weeks post-transplant (Supplementary Figure S1E). Interestingly, mice receiving Bcat1 KO ΔE-NOTCH1 GFP+ cells, although developing leukemia, showed a delay in succumbing to leukemia with respect to mice receiving Bcat1 WT ΔE-NOTCH1 GFP+ cells.

We determined the capacity of our candidate metabolites (threonine, malic acid, proline, glycine, succinic acid, and lactic acid) to correctly discriminate between neoplastic tissue, i.e., metabolically heterogeneous Δ*E-NOTCH1* tumors obtained in a Bcat1 knockout background (Bcat1^−/−^ NOTCH1-T tumors; expected to be partially metabolically different to Bcat1^+/+^ NOTCH1-T tumors), and non-neoplastic tissue (thymus). Interestingly, these metabolites were able to correctly classify all six Bcat1^−/−^ NOTCH1-T tumors as neoplastic tissue (Figure 2D), independently from the machine learning algorithm used (PLS-DA, linear support vector machine (SVM), or random forests).

### 2.2. Metabolic Profiling in Plasma

Next, we analyzed metabolic signatures in plasma from (i) leukemia-bearing mice (NOTCH1-T tumors); (ii) mice injected with Lin-negative murine hematopoietic progenitors transduced with an empty vector (MiGRI) that do not develop leukemia (non-leukemic mice (NLM), at two time points post transplantation: early (2–3 weeks) and late (8–10 weeks); and (iii) mice with an abnormal but non-tumorigenic polyclonal CD4+CD8+ DP subset (pre-leukemia) at 2–3 weeks of transplantation. We quantified 112 metabolites by using a highly sensitive capillary electrophoresis Fourier transform mass spectrometry (CE-FTMS; Ω-scan analysis) method.

**Figure 3:**
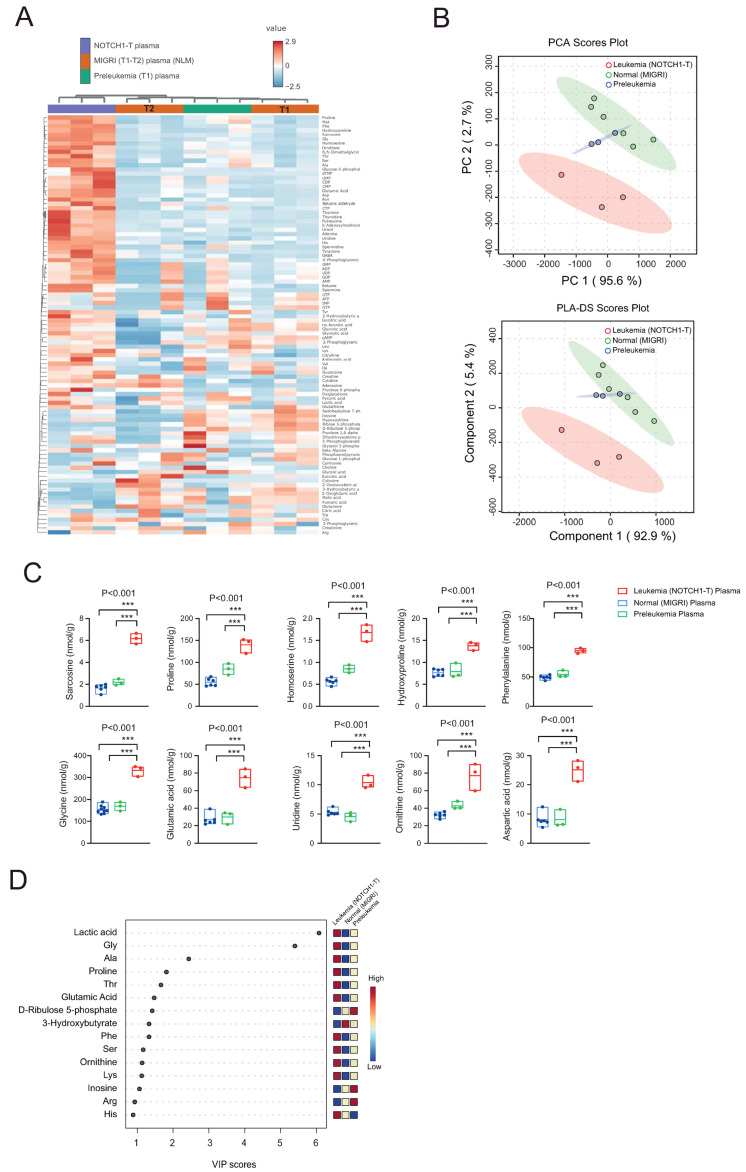
(**A**) Heat map representation of metabolites identified by capillary electrophoresis Fourier transform mass spectrometry (CE-FTMS; Ω-scan analysis) in plasma of healthy C57BL/6 mice injected with Lin-negative murine hematopoietic progenitors transduced with an empty vector (MiGRI) which do not develop leukemia (non-leukemic mice (NLM)) at two time points post transplantation: early (2–3 weeks; T1) and late (8–10 weeks; T2), mice with an abnormal but non-tumorigenic polyclonal CD4+CD8+ DP subset at 2–3 weeks of transplantation (pre-leukemia; T1) and ΔE-NOTCH1 tumor-bearing mice at moment of sacrifice (8–10 weeks). Each plasma sample represents the pooling of three different mice. (**B**) Principal component analysis (PCA; top) and partial least squares-discriminant analysis (PLS-DA; bottom) plots. (**C**) Floating bars showing Ω-scan metabolic quantification for selected metabolites differentially expressed between the plasma of NLM, preleukemic, and ΔE-NOTCH1 tumor-bearing mice. Floating bars represent min and max values; solid lines represent the mean. Significance was calculated using an unpaired *t*-test. *** *p* < 0.001. (**D**) PLS-DA variable importance in projection (VIP) score derived from metabolite profiling data from plasma of NLM, preleukemic, and ΔE-NOTCH1 tumor-bearing mice.

NLM clustered separately from NOTCH1-T-bearing mice (Figure 3A), which was also found in the PCA and PLS-DA analysis, while plasma from preleukemic mice clustered in-between NLM samples (Figure 3B). One-way ANOVA revealed 39 significant hits (Fisher’s LSD post hoc analysis and *p*-value < 0.05; Figure 3C and Supplementary Table S3). The PLS-DA VIP score resulted in 13 metabolites with a VIP score >1, including lactic acid, glycine, glutamate, and 3-hydroxybutyrate (Figure 3D).

**Figure 4:**
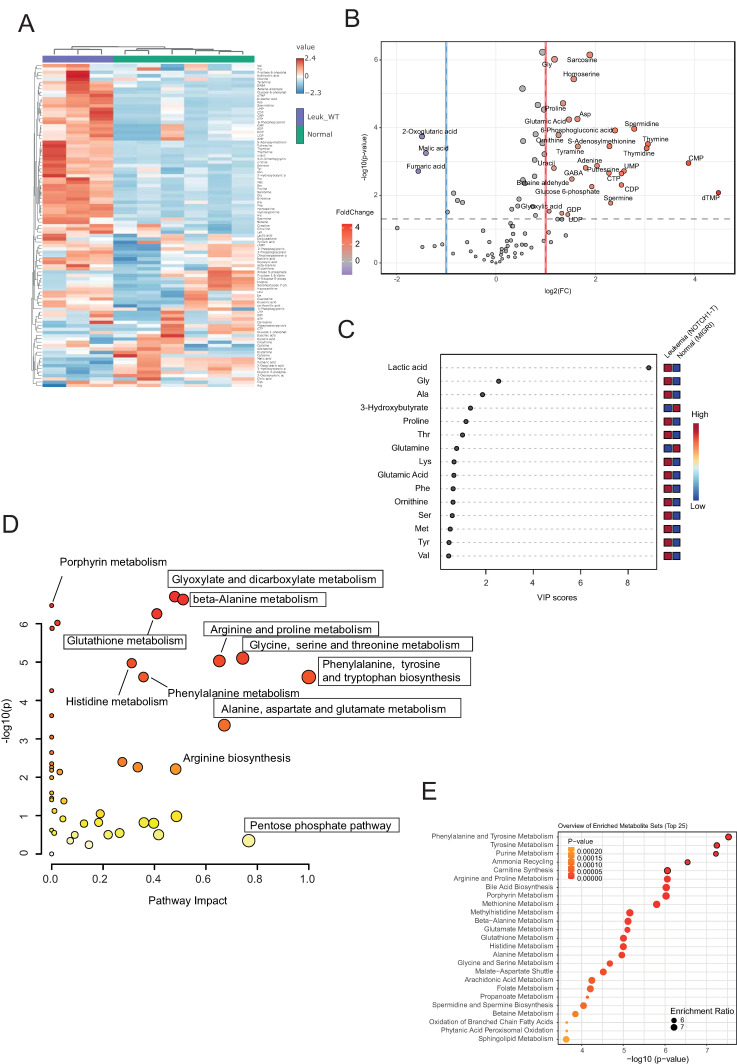
(**A**) Heat map representation of metabolites identified by capillary electrophoresis Fourier transform mass spectrometry (CE-FTMS; Ω-scan analysis) in plasma of healthy C57BL/6 mice injected with Lin-negative murine hematopoietic progenitors transduced with an empty vector (MiGRI), which do not develop leukemia (non-leukemic mice (NLM) at two time points post transplantation: early (2–3 weeks) and late (8–10 weeks), and ΔE-NOTCH1 tumor-bearing mice at moment of sacrifice (8–10 weeks). Each plasma sample represents the pooling of three different mice. (**B**) Volcano plot showing differentially expressed metabolites in plasma between NLM and ΔE-NOTCH1 tumor-bearing mice. Only metabolites with a false discovery rate (FDR)-corrected *p*-value < 0.05 and 0.5 ≤ log2 fold change (FC) ≥ 2 are shown. (**C**) PLS-DA variable importance in projection (VIP) score derived from metabolite profiling data of plasma between NLM and ΔE-NOTCH1 tumor-bearing mice. (**D**) Bubble chart depicting Metabolomics Pathway Analysis (MetPA) generated by MetaboAnalyst 6.0 software, using the KEGG database. The color of each circle represents *p*-values from pathway enrichment analysis (Y-axis; darker colors indicate more significant changes of metabolites in the corresponding pathway). On the other hand, circle size relates to the pathway impact score from pathway topology analysis (X-axis). Thus, node color and radius are based on the *p*-value and pathway impact value, respectively. (**E**) Metabolite Set Enrichment Analysis (MSEA) was used to determine differentially enriched metabolite sets between the plasma of NLM and ΔE-NOTCH1 tumor-bearing mice. The top 5 enriched pathways are highlighted with bordered circles.

We then compared plasma metabolites from NOTCH1-T versus (vs.) NLM mice to identify metabolites specifically modulated following the development of leukemia. This analysis revealed 28 upregulated and 3 downregulated metabolites (false discovery rate (FDR)-corrected *p*-value < 0.05 and 0.5 ≤ log2 fold change (FC) ≥ 2) in NOTCH1-T plasma compared to NLM plasma (Figure 4A,B and Supplementary Table S4). The PLS-DA variable importance in projection (VIP) score identified six metabolites with a VIP score > 1 (lactic acid, glycine, alanine, 3-hydroxybutyrate, proline, and threonine; Figure 4C).

To identify which metabolic pathways were altered in plasma following leukemic transformation, we performed MetPA for NOTCH1-T vs. NLM. This analysis disclosed numerous pathways to be significantly upregulated, with a high impact score in the plasma of leukemic mice compared to NLM, including phenylalanine, tyrosine, and tryptophan biosynthesis (impact score = 1.0); glycine, serine, and threonine metabolism (impact score = 0.74); alanine, aspartate, and glutamate metabolism (impact score = 0.67); glutathione metabolism (impact score = 0.40); and beta-alanine metabolism (impact score = 0.51) (Figure 4D and Supplementary Table S5).

Metabolite set enrichment analysis identified phenylalanine and tyrosine metabolism as well as purine metabolism as highly significantly enriched pathways in the plasma of NOTCH1-T-bearing mice compared to NLM (Figure 4E).

### 2.3. Biomarker Analysis in Plasma

**Figure 5:**
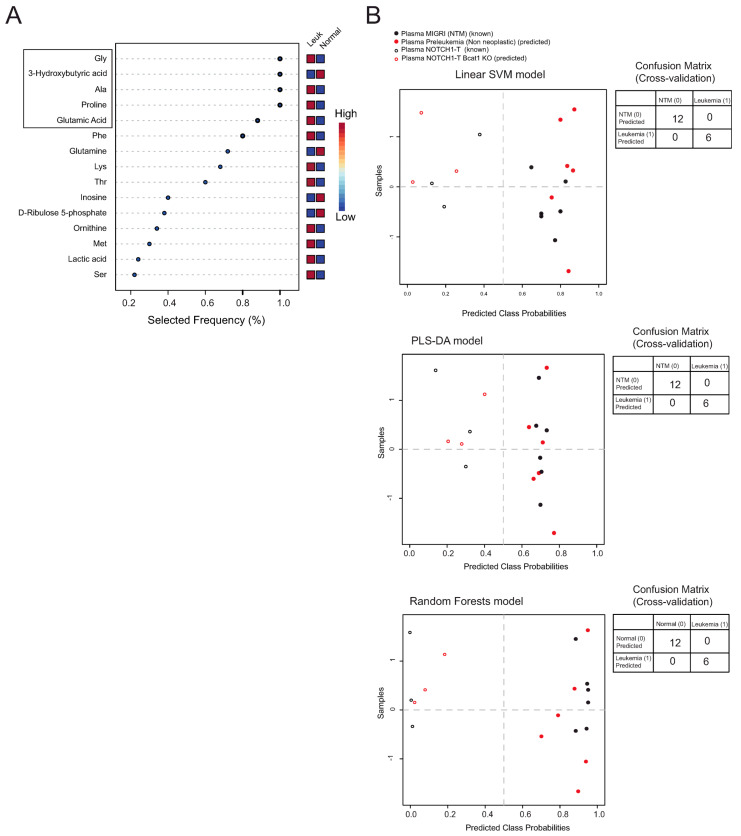
(**A**) Multivariate exploratory ROC analysis (linear SVM classification method, PLS-DA feature ranking method with 2 latent variables) was used to identify the best biomarker candidates between the plasma of NLM and ΔE-NOTCH1 tumor-bearing mice. The ranked feature details table is depicted. Box highlights best biomarker metabolites used. (**B**) Evaluation of biomarker models based on five identified biomarker metabolites (glycine, 3-hydroxybutyrate, alanine, proline, and glutamic acid) was performed using different algorithms: linear support vector machine (SVM; top); PLS-DA (middle); random forests (bottom). These models were applied to samples used for building the model (known; empty circles and full black circles) and unknown samples to predict (empty and full red circles). Averages of predicted class probabilities of each sample in the 100 cross-validations are summarized in the prediction overviews. Corresponding confusion matrices are provided next to each prediction overview.

Given the very evident differences in the metabolite signatures of plasma from leukemia-bearing mice compared to NLM, we attempted an exploratory plasma biomarker analysis to identify potential biomarkers that can distinguish between mice with leukemia and non-leukemia-bearing mice. We thus analyzed the predictive performance of the top five discriminating metabolites found using multivariate exploratory ROC analysis with the SVM classification method (four upregulated metabolites and one downregulated metabolite) in plasma from leukemia-bearing mice compared to NLM (Figure 5A). The predictive performance of our selected biomarker candidates (*n* = 5) based on the obtained area under the ROC curve (AUROC) is illustrated in Supplementary Figure S3. We then used the five biomarker candidates to evaluate their performance using well-established machine learning and statistical algorithms (SVM, PLS-DA, and random forests) in screening for leukemia on additional plasmas obtained from mice developing genetically different NOTCH1-dependent tumors (Δ*E-NOTCH1* tumors obtained in a Bcat1 knockout background; Bcat1^−/−^ NOTCH1-T tumors) and plasmas obtained from mice having an abnormal but non-tumorigenic polyclonal CD4+CD8+ DP subset (pre-leukemia) in a Bcat1^+/+^ or Bcat1^−/−^ background. The results for each five-biomarker model created using linear SVM, PLS-DA, or random forests algorithms (Figure 5B) clearly indicated a very strong discriminative capability of these five metabolites in detecting leukemia. Indeed, independently from the algorithm used, all unknown samples (9/9 plasma samples) were assigned to the correct group (non-leukemic/normal vs. leukemic).

## 3. Discussion

During tumorigenesis, cancer cells acquire hallmarks that distinguish them from their normal counterparts. One of these features is metabolic reprogramming. Leukemic cells often encounter metabolic adverse conditions such as low oxygen, glucose, and nutrient levels. This requires them to adapt metabolically in order to maintain energetic balance and continue to proliferate. A known trait of cancer cells is their reliance on aerobic glycolysis (also known as the “Warburg effect”) for energy production instead of oxidative phosphorylation (OXPHOS) [19,20]. This is in contrast to what happens in most nonmalignant cells, which rely mainly on OXPHOS to generate energy. Our metabolomics data executed on tissues confirm this notion, showing NOTCH1 T tumors manifest high levels of lactic acid (indicative of elevated glycolysis). These tumors also have high levels of TCA intermediates (succinic, malic, and fumaric acids), indicating that OXPHOS is also active. On the other hand, it seems normal thymus (>90% CD4+/CD8+ T cells) uses FAO to generate energy. This picture seems rather similar to that described between melanocytes and melanoma cell lines [21]. Interestingly, NOTCH1 T tumors present enrichment for pentose phosphate pathway and glutathione metabolism metabolites, indicating an adaptive response to counteract high levels of reactive oxygen species (ROS) necessary to maintain pro-tumorigenic signaling and resistance to apoptosis. Another interesting finding was the alteration in BCAA metabolism. In the past few years, the metabolism of BCAAs has received significant scientific interest. Notably, changes in BCAA metabolism in various tumors are principally due to increased expression of Bcat1. Our preliminary results indicate that Bcat1 may be a NOTCH1 target and that Bcat1 expression increases following leukemia development (Supplementary Figure S1A). This may explain the alterations found in BCAAs (isoleucine, valine) and BCKA (3-methyl-2-oxovalerate). Interestingly, NOTCH1-Bcat1 knock-out tumors, which may have an altered metabolism compared to Bcat1 expressing tumors, maintained the leukemic signature and could be distinguished from normal tissue. Future studies need to be conducted to elucidate the role of Bcat1 in T-ALL development.

Next, we executed targeted metabolomics on plasma from mice at different stages of leukemia development (non-leukemic, pre-leukemic, and leukemic) with the intention of identifying metabolites predictive of leukemia (leukemia biomarkers) to propose for validation in human studies. We found that NLM and mice presenting a polyclonal non-neoplastic CD4+/CD8+ population (pre-leukemia) presented a rather similar metabolic profile, which differed substantially from that seen in leukemic mice. Plasma metabolites differentially expressed between leukemic and NLM were found to be implicated in numerous pathways, including phenylalanine, tyrosine, and tryptophan biosynthesis; glycine, serine, and threonine metabolism; alanine, aspartate, and glutamate metabolism; glutathione metabolism; and beta-alanine metabolism. Of these, beta-alanine metabolism has recently been described to be increased in melanoma-bearing mice compared to healthy controls [22], while glutamine metabolism has previously been shown to be altered in ALL patients [23]. Our proposed metabolic biomarkers (threonine, malic acid, proline, glycine, succinic acid, lactic acid, 3-hydroxybutyrate, alanine, and glutamic acid) are conserved between mice and humans and play fundamental roles in central metabolic pathways that are highly conserved across mammalian species (Warburg effect, TCA cycle, and amino acid metabolism). Indeed, a recent study using untargeted metabolomics between ALL patients and healthy donors [24] found alterations in many pathways in common with us, suggesting that it is likely that the differences we found using a murine model of T-ALL could apply to a human setting.

Based on our findings in plasma, we propose five metabolites as potential leukemia biomarkers, including glycine, 3-hydroxybutiric acid (3-HB), alanine, proline, and glutamic acid, to distinguish healthy from leukemia-bearing mice. Of these, 3-HB seems to be an interesting metabolite. Indeed, 3-HB is a ketone body and serves as an alternative energy source for tissues when glucose levels are low, thus potentially affecting tumor growth. Indeed, in cancer, the role of 3-HB is complex and is still being elucidated [25]. We find low levels of 3-HB in the plasma of leukemic mice compared to NLM (while in NOTCH1 T tumors it seems to accumulate, *p* = 0.07), similarly to another recent study [23], suggesting increased uptake/utilization of ketone bodies in cancer. Future studies are warranted to establish the role of ketone bodies in leukemia progression.

Although our study has several limitations (one model of T-ALL, NOTCH1 dependent T-ALL, limited number of samples, targeted metabolomics), the reduced number of confounding factors in our model, which may influence serum/plasma metabolomics in human samples (disease stage, gender, drug intake, and environmental factors), may help in better identifying the real metabolic differences between leukemia patients and healthy controls. However, validating mouse metabolic biomarkers for cancer in humans is complex and challenging. Future studies will need to be performed to evaluate the validity of our proposed biomarkers for leukemia. This could be attempted through the use of humanized models (patient-derived xenografts) in immunodeficient mice or using samples (fresh tissue and plasma) in prospective (or retrospective) collaborative studies from newly diagnosed leukemia patients at diagnosis and age–sex-matched healthy individuals.

In conclusion, our results may provide useful clues for developing non-invasive assays evaluating specific metabolites for diagnosis, prognosis, or metabolic-oriented therapy in NOTCH1-dependent leukemias (especially T-ALL).

## 4. Materials and Methods

### 4.1. Generation of Global Bcat1^−/−^ Mice

Bcat1 knockout (^−/−^; KO) mice on a C57BL/6J background were generated using the CRISPR/Cas9 technology by Cyagen (Santa Clara, CA, USA). Briefly, the mouse Bcat1 gene (NCBI Reference Sequence: NM_001024468; Ensembl: ENSMUSG00000030268) is located on mouse chromosome 6 and consists of 11 exons (transcript: ENSMUST00000032402). Exon 6 was selected as the knockout region. F0 founder animals were identified by PCR followed by sequence analysis, and they were bred to wild-type (^+^/^+^; WT) mice to test germline transmission and F1 animal generation. Heterozygous mice were intercrossed to generate homozygous mice. Genotyping was performed by PCR using DNA isolated from peripheral blood cells and primers 1 (F1: 5′-TTCACTCTTCCTGTGAGCAGTTT-3′; R1: 5′-ACATCTCAAACCCTCTTTTGTTTCC-3′) and primers 2 (F2: 5′-GTGTTAGTTGTTGGAGGTGTTGC-3′; R1: 5′-ACATCTCAAACCCTCTTTTGTTTCC-3′).

### 4.2. Animals and Sample Collection

NOTCH1-induced T-ALL tumors were generated in mice as previously described [13]. Briefly, bone marrow (BM) cells were collected from 6- to 12-week-old WT and Bcat1 KO C57BL/6 mice, and BM progenitors (Lin-) were purified by negative selection using magnetic sorting (Miltenyi Biotec, Bergisch Gladbach, Germany). The cells were cultured overnight in the presence of the following cytokines (all from Peprotech, London, UK): mIL-3 (10 ng/mL), mIL-6 (10 ng/mL), mFLT3L (50 ng/mL), mIL7 (100 ng/mL), and mSCF (50 ng/mL). The cells were then washed, resuspended in retroviral supernatant (∆E-NOTCH1 or MIGRI empty vector), placed in the same cytokine cocktail containing polybrene (4 µg/mL), and centrifuged at 1,290g for 90 min. A second round of spinoculation was performed the following day. After flow cytometric analysis of transduced progenitors, approximately 50 × 10^4^ Lin-/Sca1+/GFP+ cells of each genotype were injected i.v. into lethally irradiated (9 Gy) recipients (6–8-week-old C57BL/6 female mice). At least two independent transplantation experiments were performed. Mice were bled after 2–3 weeks to monitor engraftment and evaluate the presence of circulating immature T cell progenitors by flow cytometry. Tumor-bearing mice were euthanized, and spleens (primary tumors) were snap frozen. For metabolomics analysis, blood was taken at the indicated time points (2–3 weeks post-transplantation (T1), 8–10 weeks post-transplantation (T2)) by submandibular puncture and collected in Eppendorf tubes containing 0.5 M EDTA. Samples were then processed according to Human Metabolome Technologies methods (HMT, Tokyo, Japan). Briefly, tubes were centrifuged for 10 min at 1200× *g* and stored at −80 °C until shipping. To better represent leukemia/control heterogeneity in the cohort of mice, each individual plasma sample analyzed represents the pool of three different mice. Procedures involving animals and their care conformed with institutional guidelines and were authorized by local (OPBA) and national (Italian Ministry of Health) animal ethical committees.

### 4.3. Western Blotting

Aliquots of frozen tissues were homogenized on ice using RIPA lysis buffer supplemented with phosphatase inhibitor cocktail sets I and II (Sigma-Aldrich, Merck, Darmstadt, Germany) and protease inhibitor cocktail tablets (Roche, Burgess Hill, UK) and normalized for protein concentration using the BCA method (Pierce, Pero, Italy). For Western blotting, protein samples were separated on 4–12% gradient Tris-Glycine SDS-PAGE Gels (Invitrogen, Thermo Fisher Scientific, Waltham, MA, USA) and transferred to PVDF membrane (Millipore, Burlington, MA, USA). The membranes were then probed with antibodies against Bcat1, total histone H3 (Cell Signaling Technology; Danvers, MA, USA), and a polyclonal anti-α-tubulin antibody from Proteintech Europe (Manchester, UK).

### 4.4. Steady State Metabolite Profiling

For in vivo experiments, thymic tissue from 6-week-old normal and Bcat1 KO C57/BL6 mice (n = 3) was obtained. Flash-frozen thymuses were subsequently analyzed by capillary electrophoresis time-of-flight mass spectrometry (CE-TOFMS; C-scope analysis, HMT, Tokyo, Japan). We also analyzed flash-frozen tissue (spleens) from ΔE-NOTCH1 leukemia-bearing mice with WT and Bcat1 KO genotypes by CE-TOFMS (C-scope analysis, HMT, Tokyo, Japan). Different NOTCH1 T tumors from independent transplantation experiments with similar immunophenotypes were analyzed. Identification of known chemical entities was based on comparison to metabolomics library entries of purified standards and was performed by HMT. A heatmap representation of the metabolites identified was performed using MetaboAnalyst [26].

### 4.5. Sample Preparation and Targeted Metabolite Profiling in Plasma

Each 50 μL plasma sample was mixed with 200 μL of methanol containing internal standards (2 μM) and mixed. Then, 150 μL of Milli-Q water was added and mixed thoroughly. The solution was filtrated at 4 °C through a 5-kDa cut-off filter (ULTRAFREE-MC-PLHCC, Human Metabolome Technologies (HMT), Yamagata, Japan) to remove macromolecules. The filtrate was centrifugally concentrated and resuspended in ultrapure water for metabolome analysis. Metabolome analysis was performed on mouse plasma using capillary electrophoresis Fourier transform mass spectrometry (CE-FTMS) in two modes for cationic and anionic metabolites (Ω-scan analysis, HMT, Tokyo, Japan); 550 metabolites (307 metabolites in cation mode and 243 metabolites in anion mode) were detected on the basis of HMT’s standard library. Among the target metabolites, 102 metabolites (53 in cation and 49 in anion mode, respectively) were detected and quantified.

### 4.6. Metabolomics Data Processing and Statistical Analysis

Data from C-scope and Ω-scan analyses were obtained from HMT, and initial data cleaning was performed by excluding metabolites with >20% missing values or values below the LOD in all experimental groups. Thus, all metabolites with >80% of the concentration values above the LOD in at least one of the experimental groups were included for statistical analysis. The remaining missing values were replaced by 1/5 of the minimum positive value of each variable. Data were then analyzed using the web-based tool MetaboAnalyst 6.0 (https://www.metaboanalyst.ca) [26]. For both tissue and plasma data, significant changes in metabolite levels were identified by a one-way ANOVA followed by Fisher′s LSD post hoc test. To correct for multiple comparisons and thus minimize false positives, FDRs were calculated based on the Benjamini–Hochberg procedure. FDR-corrected *p*-values < 0.05 were considered statistically significant. For plasma and tissue data, FCs were calculated to evaluate differences between metabolites obtained from leukemia-bearing mice compared to non-leukemia mice. FCs > 2.0 were considered important. Both unsupervised PCA and supervised PLD-DA were performed whenever necessary to determine the metabolic signature contributing to group separation. PLS-DA is prone to data overfitting; thus, the quality of the model was assessed by cross-validation (calculation of Q2, R2, and accuracy values), and the overfitting tendency of the model was validated using permutation testing. The PLS-DA VIP score was calculated, and metabolites with a VIP score > 1 were considered important for group separation. The Mus musculus KEGG pathway libraries were used as references for the MetPA or enrichment analysis of leukemia tissue metabolites and mouse plasma metabolites. For exploratory biomarker analysis, multivariate exploratory ROC analysis (linear SVM classification method, PLS-DA feature ranking method with 2 latent variables) was used to identify the best biomarker candidates between leukemia-bearing mice and healthy controls. Heatmaps and Venn diagrams were created using MetaboAnalyst 6.0.

### 4.7. Flow Cytometry Analysis

Peripheral blood (PB) and spleens were harvested from WT and KO mice. Red blood cell (RBC) lysis was performed using a hypotonic solution containing ammonium chloride for all samples. Briefly, the cells were blocked for 10 min with CD16/CD32 (mouse BD FC Block, BD Pharmingen, Oxford, UK) diluted 1:100 in PBS at 4 °C and subsequently stained for 30 min with a combination of the following panel of antibodies: Cd3e-BV421/BV510 (Biolegend, London, UK), Cd8-BV605/PE, and Cd4-FITC/APC (all from BD Pharmingen). The fixable viability stain dye (FVS780; BD) was used to analyze only viable cells. Cells were analyzed on a BD LSR II flow cytometer, and acquired data was analyzed with FlowJo (Tree Star Inc., Ashland, OR, USA).

### 4.8. Statistical Analysis

Student’s *t*-tests and ANOVA tests were used where appropriate. All statistical tests were two-sided and unpaired, and *p* < 0.05 was considered statistically significant (* *p* < 0.05, ** *p* < 0.01, *** *p* < 0.001). Statistical analyses were performed with GraphPad Prism software version 8.4.3 (GraphPad Software, San Diego, CA, USA).

## Data Availability

The data presented in this study are available on request from the corresponding author.

## Supplementary Materials

The following supporting information can be downloaded at https://www.mdpi.com/article/10.3390/ijms25126543/s1.
