# Supplementary material for: Targeted Metabolomics of Tissue and Plasma Identifies Biomarkers in Mice with NOTCH1-Dependent T-Cell Acute Lymphoblastic Leukemia
Source: Int J Mol Sci. 2024 Jun 13;25(12):6543. doi: 10.3390/ijms25126543 (PMC11204162; doi:10.3390/ijms25126543)
Supplement: Supplementary file 1 [file ijms-25-06543-s001.zip › ijms-3022219-supplementary_EP.pdf]

Supplementary Table S1: Differentially expressed metabolites in NOTCH1-T tumors compared to thymic tissue.

Supplementary Table S2: Metabolic pathways altered following leukemic transformation using MetPA.

Supplementary Table S3: Differentially expressed metabolites in plasma of healthy C57BL/6 mice injected with Lin-negative murine hematopoietic progenitors transduced with an empty vector (MiGRI) which do not develop leukemia (non-leukemic mice (NLM), mice with an abnormal but non-tumorigenic polyclonal CD4+CD8+ DP subset at 2-3 weeks of transplantation (pre-leukemia) and ΔE-NOTCH1 tumor bearing mice at moment of sacrifice.

Supplementary Table S4: Differentially expressed metabolites in plasma of healthy C57BL/6 mice injected with Lin-negative murine hematopoietic progenitors transduced with an empty vector (MiGRI) which do not develop leukemia (non-leukemic mice (NLM)) and ΔE-NOTCH1 tumor bearing mice at moment of sacrifice.

Supplementary Table S5: Metabolic pathways altered in plasma following leukemic transformation using MetPA.

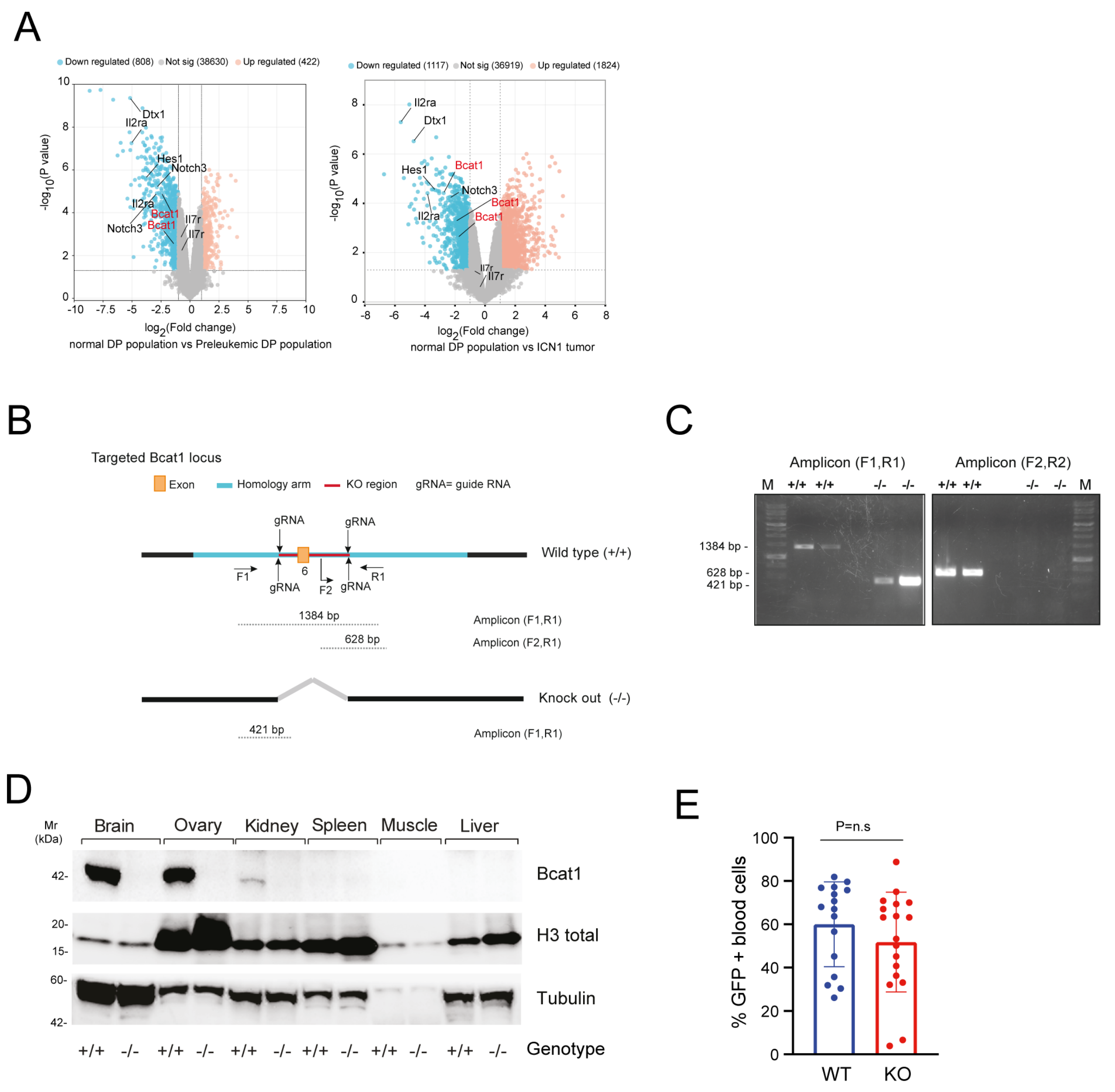

Supplementary Figure S1. Bcat1 is upregulated following leukemia development and generation of Bcat1 knock-out (KO) mouse. (**A**) Volcano plot showing differentially expressed genes between normal double positive (DP) cells and the pre-leukemic DP population (left panel) and between normal DP cells and ICN1-induced DP leukemic cells (right panel) from GSE12948. The probes for some known NOTCH1 targets are highlighted in black. The probes for Bcat1 are shown in red. (**B**) Gene targeting strategy for the *Bcat1* locus using the CRISPR/Cas9 system. (**C**) PCR genotype analysis. Gel electrophoresis image showing PCR products amplified from WT (n=2) or Bcat1-/- (n=2) mice using primers reported in Materials and methods section. M: molecular marker. (**D**) Immunoblots of Bcat1 in selected organs from WT and Bcat1 null mice. Total histone H3 and α-tubulin are shown as loading controls. (**E**) Lethally irradiated mice were reconstituted with donor bone marrow cells (WT or KO for *Bcat1*) transduced with Δ*E-NOTCH1* allele or GFP alone (MigR1; data not shown). GFP percentages in the blood (PB) are shown at early (2-3 weeks) after bone marrow transplant (BMT). These represent pre-leukemic cells. Significance was calculated using an unpaired two-tailed t-test. n.s.= not significant.

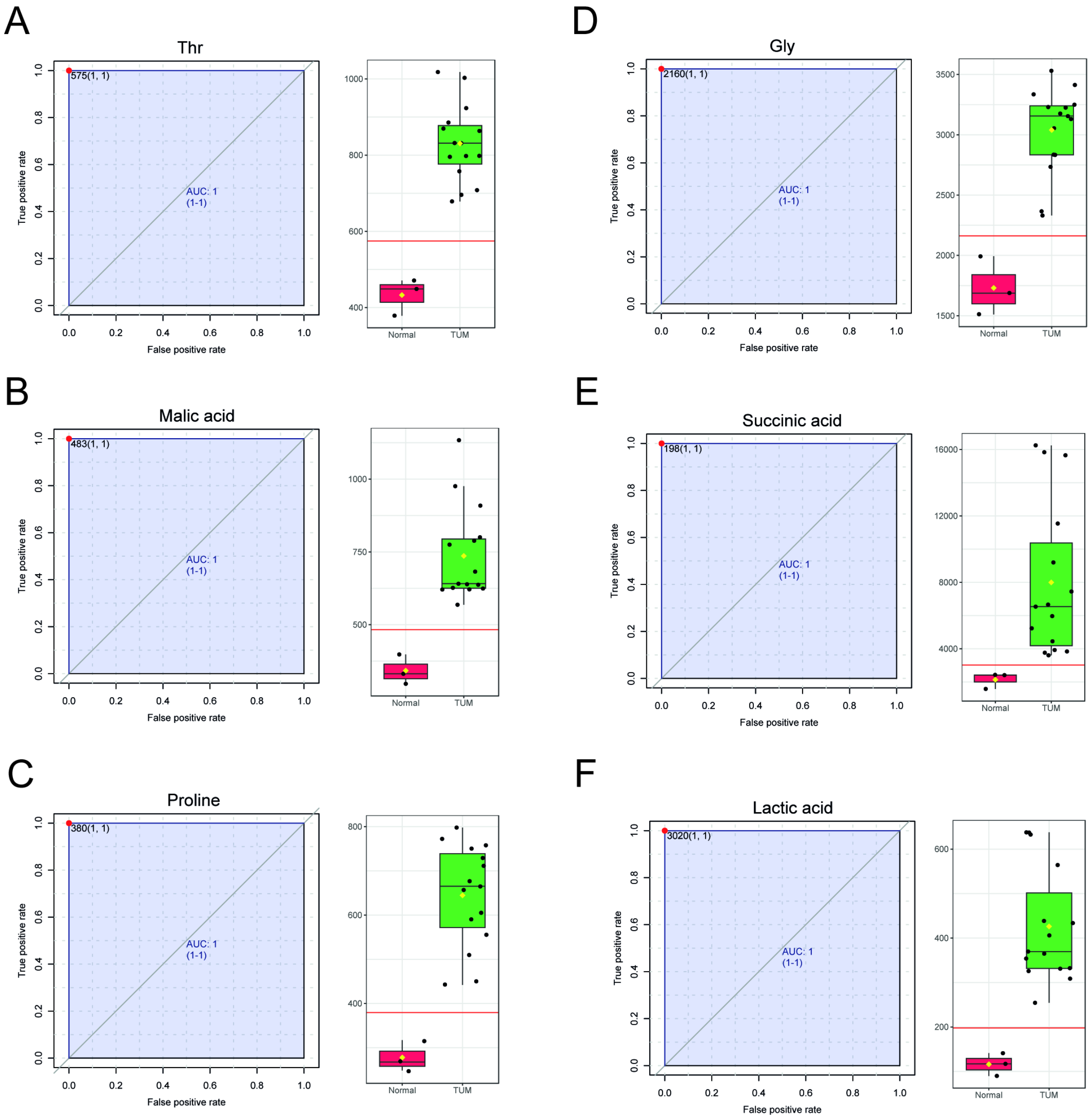

Supplementary Figure S2. Predictive performance of potential leukemia biomarker candidates (tissue). Individual ROC curve analysis (left) and expression box plots (right) of (**A**) threonine (Thr), (**B**) malic acid, (**C**) proline, (**D**) glycine (Gly), (**E**) succinic acid, (**F**) lactic acid. Evaluation is based on the comparison of metabolite concentrations in tissue samples obtained from healthy thymus (Normal; *n*=3) versus splenic leukemic tissue (TUM; *n*=15).

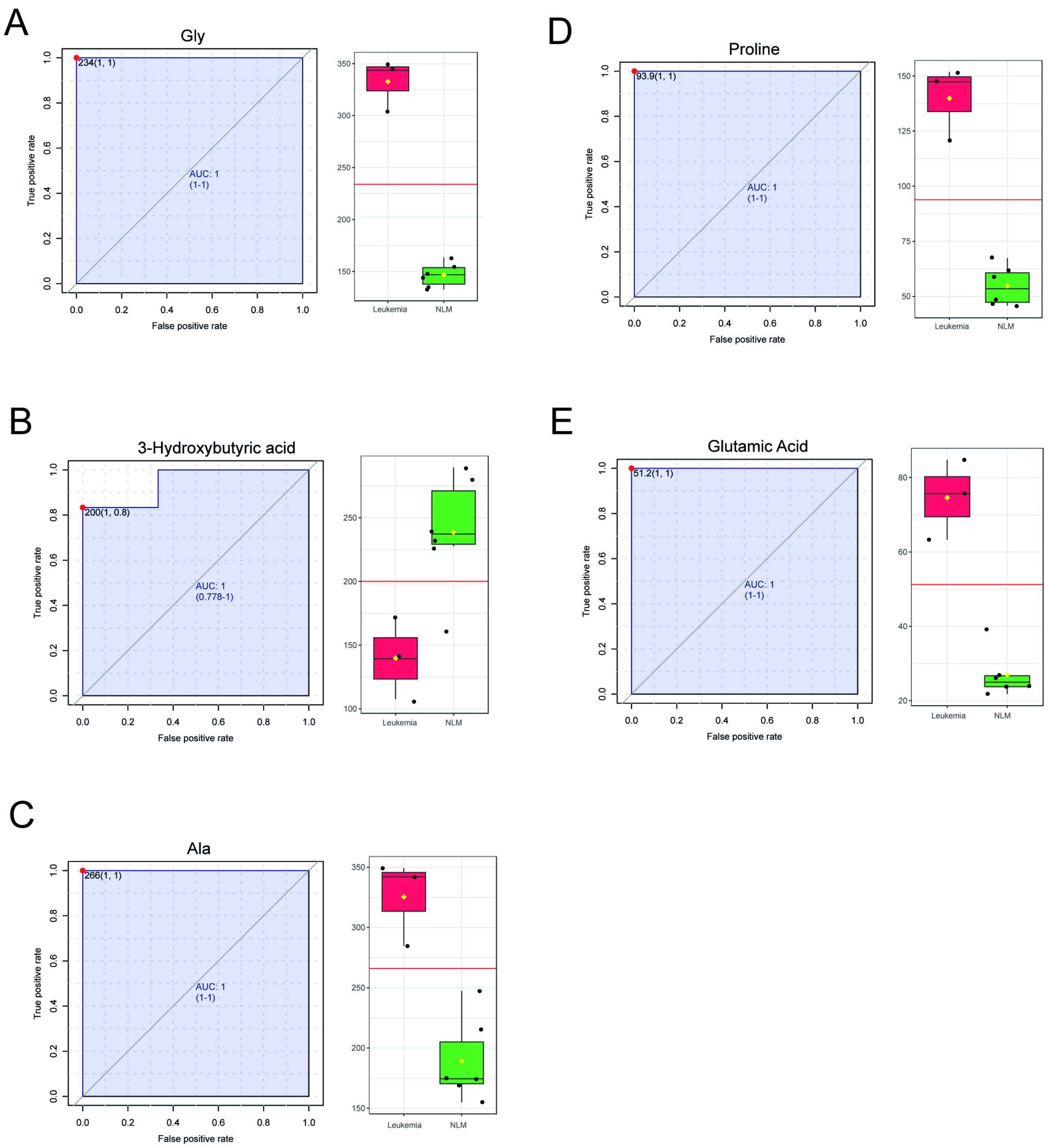

Supplementary Figure S3. Predictive performance of potential plasma leukemia biomarker candidates. Individual ROC curve analysis (left) and expression box plots (right) of (**A**) glycine (Gly), (**B**) 3-hydroxybutyrate, (**C**) alanine (Ala), (**D**) proline, (**E**) glutamic acid. Evaluation is based on the comparison of metabolite concentrations in plasma samples obtained from non-leukemic mice (NLM) (NLM; *n*=6) versus leukemic mice (Leukemia; *n*=3).
