# Supplementary figures and images for: Targeted Metabolomics of Tissue and Plasma Identifies Biomarkers in Mice with NOTCH1-Dependent T-Cell Acute Lymphoblastic Leukemia
Source: Int J Mol Sci. 2024 Jun 13;25(12):6543. doi: 10.3390/ijms25126543 (PMC11204162; doi:10.3390/ijms25126543)

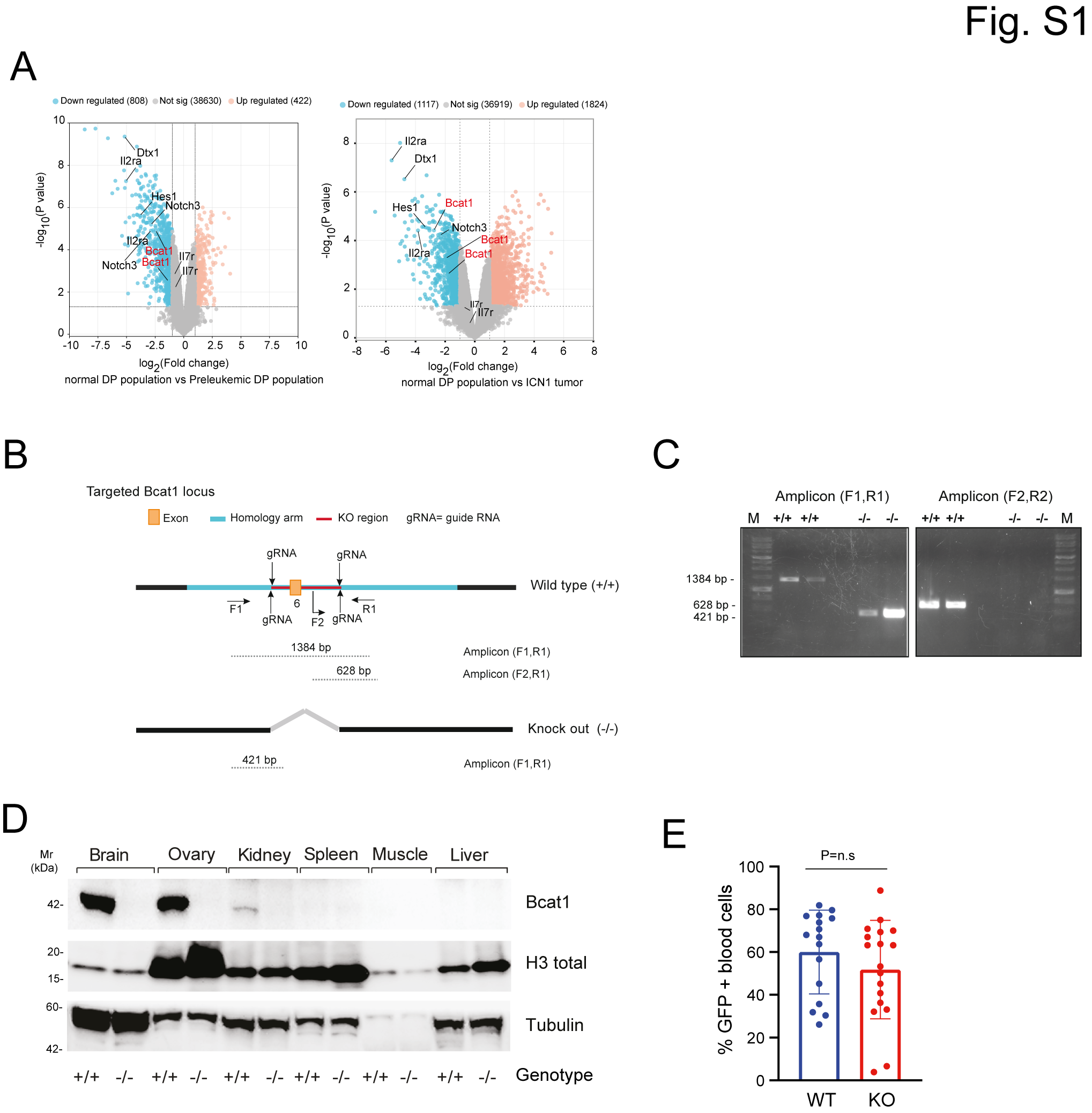

Supplement: Supplementary file 1 [file ijms-25-06543-s001.zip › Figure S1REV.tif]

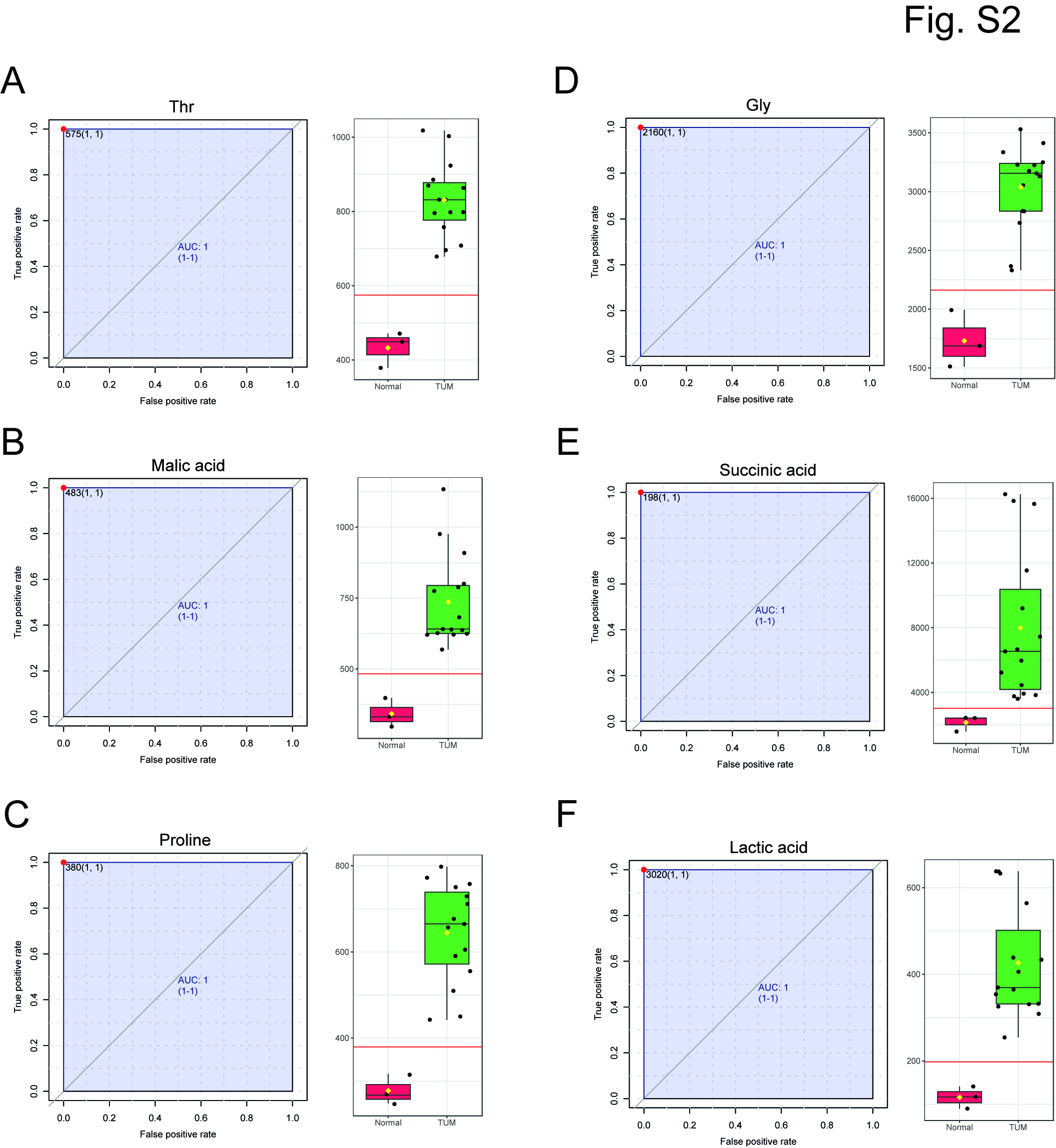

Supplement: Supplementary file 1 [file ijms-25-06543-s001.zip › Figure S2REV.tif]

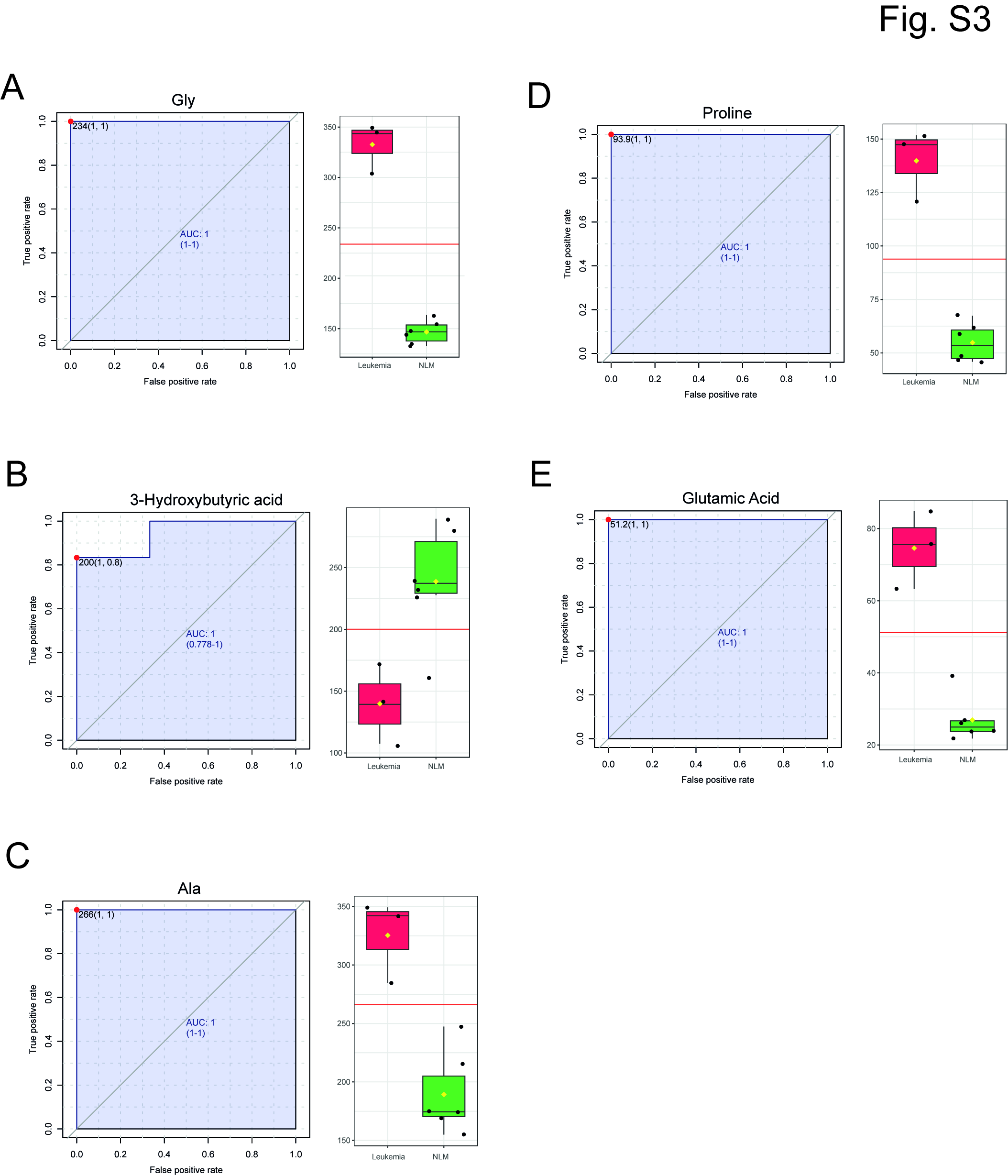

Supplement: Supplementary file 1 [file ijms-25-06543-s001.zip › Figure S3REV.tif]
